# New perspectives on fertility in transwomen with regard to spermatogonial stem cells

**DOI:** 10.1530/RAF-22-0022

**Published:** 2023-01-18

**Authors:** Jennifer Dabel, Florian Schneider, Joachim Wistuba, Sabine Kliesch, Stefan Schlatt, Nina Neuhaus

**Affiliations:** 1 Institute of Reproductive and Regenerative Medicine, Centre of Reproductive Medicine and Andrology, Muenster, Germany; 2 Department of Clinical Andrology, Centre of Reproductive Medicine and Andrology, Muenster, Germany

**Keywords:** spermatogonia, gender confirming hormone therapy, spermatogonesis, transwomen, fertility preservation

## Abstract

**Objective:**

Germ cells of transwomen are affected by gender-affirming hormone therapy (GAHT). Fertility will be lost after surgical intervention; thereby, fertility preservation becomes an increasingly imortant topic. This study investigated if the absolute number of spermatogonia in transwomen is comparable at the time of gender-affirming surgery (GAS) to that in pre-pubertal boys.

**Methods:**

We carried out a retrospective study of testicular tissues from 25 selected subjects, which had undergone a comparable sex hormone therapy regimen using cyproterone acetate (10 or 12.5 mg) and estrogens. As controls, testicular biopsies of five cisgender adult men (aged 35–48 years) and five pre-/pubertal boys (5–14 years) were included. Testicular tissues were immunohistochemically stained for MAGE A4-positive cells, the most advanced germ cell type. The number of spermatogonia per area was assessed. Clinical values and serum hormone values for FSH, LH, testosterone, free testosterone, estradiol and prolactin were determined on the day of GAS for correlation analyses.

**Results:**

Round spermatids were the most advanced germ cell type in 3 subjects, 5 had an arrest at spermatocyte stage, while 17 showed a spermatogonial arrest. On average, testicular tissues of transwomen contained 25.15 spermatogonia/mm^3^, a number that was significantly reduced compared to the two control groups (*P* < 0.01, adult 80.65 spermatogonia/mm^3^ and pre-/pubertal boys 78.55 spermatogonia/mm^3^). Linear regression analysis revealed that testes with higher weight and high LH contained more spermatogonia.

**Conclusion:**

Irrespective of treatment dose or duration, spermatogenesis was impaired. Spermatogonial numbers were significantly reduced in transwomen compared to the control groups.

**Lay summary:**

When transwomen go through treatment to confirm their gender, their germ cells are affected. They lose their fertility after surgery, so fertility preservation becomes an important topic. We carried out a study looking at tissue from testes of 25 people who had been through the same sex hormone therapy until surgery. Blood samples were also taken. As controls, samples were taken from the testes of cisgender boys and adult men. On average, the samples from the testes of transwomen contained a smaller number of early sperm cells compared to the two control groups. Regardless of the dose or length of hormone treatment, the fertility of transwomen was significantly reduced so that counseling about fertility preservation should be offered before hormone therapy.

## Introduction

Fertility preservation is recommended for patients who are at risk of infertility due to disease or gonadotoxic treatment. After the onset of puberty and initiation of spermatogenesis, cryopreservation of sperm can be offered as a validated clinical procedure to men. In the case of azoospermia, sperm can be directly retrieved from the testes using testicular sperm extraction (TESE,Tüttelmann *et al.* 2011). For pre-pubertal patients, however, in whom spermatogenesis has not yet been initiated, specialized centers worldwide offer cryopreservation of immature testicular tissues that contain population of diploid spermatogonia, including the spermatogonial stem cells (SSCs), which form the basis for initiation of spermatogenesis later in life (Goossens *et al.* 2020). Spermatogonia are located at the basal membrane of the seminiferous tubules and differentiate as interconnected chains of cells, which can give rise to meiotic spermatocytes and ultimately to haploid spermatids. After spermiogenesis, spermatids are released into the lumen of seminiferous tubules as immotile spermatozoa (Weinbauer *et al.* 2010). Also, spermatogonia have the ability to undergo self-renewing divisions thereby maintaining the stem cell pool. Specific cryopreservation protocols to preserve spermatogonia, which are different from established protocols to cryopreserve sperm, have been developed (Goossens *et al.* 2020). It is of note that protocols to derive sperm from these undifferentiated germ cells remain to be established. The cryopreservation of immature testicular tissues can in principle also be considered for younger trans-females starting gender-affirming hormone therapy (GAHT) and adult men showing regressed spermatogenesis following GAHT. The spermatogonial numbers in these tissues compared to other immature tissues in the fertility preservation cohorts remain largely unknown.

In principle, three approaches are under development which are autologous germ cell transplantation, autologous grafting of testicular tissues into the scrotum and *in vitro* spermatogenesis (Medrano *et al.* 2018, Sharma *et al.* 2019). As the former two approaches require male gonads to achieve spermatogenesis, in particular, the approach of *in vitro* spermatogenesis may perspectively be suitable for transwomen. As the differentiation efficiencies in model organisms with normal spermatogonial numbers are reportedly low (Heckmann *et al.* 2018), information on the spermatogonial numbers in transwomen at the time of GAS are highly relevant.

Transwomen undergo GAHT prior to gender-affirming surgery (GAS) for physical adaptation to the female body. Generally, GAHT has a negative influence on steroidogenesis as well as spermatogenesis in transwomen. In Germany, GAHT usually consists of a combination of anti-androgens such as cyproterone acetate (CPA) and estrogens (Schneider *et al.* 2019). This combination leads to low levels of luteinizing hormone (LH) and follicle-stimulating hormone (FSH). The intake of estrogens promotes the synthesis of prolactin (De La Torre *et al.* 1978, Labrie *et al.* 2005). CPA treatment results in decreased ejaculate volume, decreased sperm number and impaired motility and morphology, after 2–4 months (Føgh *et al.* 1979, Wang & Yeung 1980).

GAS constitutes the final step toward the physical adaptation to the female body. Histological analyses of testicular tissues obtained in this process revealed intact spermatogenesis only in a limited number of individuals (Schneider *et al.* 2015, Leavy *et al.* 2017, Jindarak *et al.* 2018, Kent *et al.* 2018). In the majority of testicular tissues of transwomen, spermatogenesis was suppressed and spermatogonia were the most advanced germ cell type that remained (Lu & Steinberger 1978, Schulze 1988, Venizelos & Paradinas 1988, Kisman *et al.* 1990, Schneider *et al.* 2015, Jindarak *et al.* 2018, Matoso *et al.* 2018). As GAS involves testes removal and thereby leads to irreversible infertility, early counseling with regard to fertility preservation options is of utmost importance according to the national and international guidelines (AWMF Guideline (available at https://www.awmf.org/leitlinien/detail/ll/138-001.html), WPATH guideline (Coleman *et al.* 2012)). While the demand for fertility preservation in transwomen has been reportedly low (Wierckx *et al.* 2012, Auer *et al.* 2018, Riggs *et al.* 2018), almost 40% of participants in respective surveys stated that they would have considered preservation of germ cells if they had been counseled accordingly (Wierckx *et al.* 2012).

Provided that the spermatogonial quantity is sufficient, these individuals could be included into fertility preservation programs that have been established for pre-pubertal boys (Goossens *et al.*2020). Importantly, cryopreservation of immature testicular tissues could be an option for transgirls who start GAT already prior to puberty (De Roo *et al.* 2016). To date, information on spermatogonial numbers in testicular tissues of transwomen are limited. Therefore, the aim of this study was to assess spermatogonial numbers in transwomen at the time of GAS in comparison to adult and pre-/pubertal patients.

## Materials and methods

### Ethical approval

Testicular tissue collection of transwomen was approved by the ethical committee of the Ärztekammer Westfalen-Lippe (No. 2012-555-f-S) and the Ärztekammer Hamburg (No. MC-131/13). Ethical approval for testicular biopsies of male infertility patients with obstructive azoospermia was given by the Ethics Committee of the Medical Faculty of Münster and the State Medical Board (No. 2011-520-f-S). Testicular tissues of pre-/pubertal boys were obtained in the frame of ‘Androprotect’, a German fertility preservation program. The testicular tissue retrieval was approved by the Ethics Committee of the Medical Faculty of Münster and the State Medical Board (No. 2011-520-f-S). Informed and written consent was obtained from all subjects prior to surgery.

### Included cohort

For this study, tissues from transwomen (*n* = 25) were used that had been treated with a similar dose of CPA, 10 or 12.5 mg (mean treatment duration: 27.6 months, range: from 11 to 66 months), which is still the most prevelant way of treating transwomen in Germany. Testes of the 25 transwomen (mean age: 28.1 years, range: from 16 to 40 years) were removed on the day of GAS which were performed in three clinics with different treatment strategies. Clinic A (*n*  = 5) stopped the GAHT 4–6 weeks prior to the surgery and clinic B (*n* = 16) stopped the hormone therapy 2 weeks prior to the surgery, while clinic C (*n* = 4) continued the therapy until the day of GAS. For 19 persons, the estrogen consumption was reported (mean: 3.9 mg/day, range: 0–8 mg/day). As controls, we used testicular tissue samples from two men with obstructive azoospermia and three men with an obstruction due to a CFTR mutation presenting at the Center of Reproductive medicine and andrology (mean age: 38.8 years, range: between 35 and 48 years). Qualitatively intact spermatogenesis of adult men was demonstrated by routine histological analyses which revealed germ cell differentiation to elongated spermatids in more than 75% of the seminiferous tubules. Moreover, testicular tissues from five pre-/pubertal boys (mean age: 9.8 years, range: from 5 to 14 years) were selected, whose tissues were collected within the frame of the German fertility preservation network Androprotect. The testicular tissues were unaffected by disease or treatment. The control groups are not age-matched to the transgender subjects.

### Hormone measurements

Heparinized and EDTA blood samples were taken prior to or on the day of GAS. Serum values of FSH, LH, testosterone, free testosterone, estradiol and prolactin were measured on an Abbot Architect i1000SR automated analyzer (Abbott Diagnostics) with the chemiluminescent microparticle immunoassay according to published protocols (Ragab *et al.* 2018). Before the hormonal measurements were performed, commercially available internal quality controls were run (Lyphochek® Immunoassay Plus Control Trilevel 370; BioRAD and ARCHITECT SHBG Controls; 08K26-10, Wiesbaden, Germany) as part of the routine instrument operation.

### Human testicular tissue and preparation

The testes of transwomen were transported to the CeRA in 1×× PBS or DMEM (Life Technologies) at 4°C. Testes weight was determined with and without testicular capsule and considered to reflect testicular volume (Behre *et al.* 1989). Testis size of pre-/pubertal and adult patients was assessed by ultrasound and testicular volume was calculated using the previously published method.

Testicular tissue fragments from transwomen as well as controls were fixed overnight in Bouin’s solution. Tissues were routinely embedded in paraffin using a Histomaster machine (Histomaster Modell 2080/T, Enno Vieth, Wiesmoor, Germany) and sectioned at 3 µm (Leica SM 2000R microtome, Leica Microsystems GmbH).

### Histological and immunohistological staining of testicular tissue

For overview staining and evaluation of the most advanced germ cell type, sections were deparaffinized, dehydrated and periodic acid-Schiff (PAS)-stained according to previously published protocols (Brinkworth *et al.* 1995). The sections were counterstained with Mayer’s hematoxylin (Mayer’s hemalaum solution; 1.09249.0500; Merck Millipore), dehydrated and mounted in Merckoglas (103973.0001; Merck Millipore).

The protocol for immunohistochemical stainings was performed as previously published (Albert *et al.* 2012). Briefly, the sections were incubated overnight at 4°C with the primary antibody MAGE A4 (melanoma-associated antigen 4, provided by Prof G. C. Spagnoli from University Hospital of Basel, Switzerland; dilution 1:20) which represents a marker for spermatogonia and primary spermatocytes (Lim *et al.* 2012, Kossack *et al.* 2013, Pohl *et al.* 2019). Incubation with unspecific immunoglobulin G (IgG) and omission of primary antibody served as negative controls. The sections were incubated with the respective secondary biotin-labeled antibody (goat-anti-mouse IgG biotin, Ab5886, Abcam; dilution 1:100) for 1 h at room temperature. Subsequently, horse-radish peroxidase-conjugated streptavidin (S5512, Sigma–Aldrich; dilution 1:500) was applied and sections were incubated for 45 min at room temperature. For visualization, 3,3’-diaminobenzidine tetrahydrochloride (DAB) was used as substrate. Counterstaining was performed with Mayer’s hematoxylin (Mayer’s hemalaum solution; 1.09249.0500; Merck Millipore). After dehydration, the sections were mounted with Merckoglas (103973.0001; Merck Millipore).

Images were acquired by scanning the entire tissue section using the PreciPoint (PreciPoint GmbH, Freising, Germany) microscope equipped with a 60× objective and analyzed using the ViewPoint software (PreciPoint GmbH). All histological evaluations were performed by one observer using a random-systematic sampling approach in a blinded fashion.

### Evaluation of the most advanced germ cell type

The spermatogenic state was assessed in a PAS-stained testicular tissue section. Specifically, within each cross-section, tubules were categorized according to the presence of the most advanced germ cell type (elongated spermatids, round spermatids, spermatocytes, spermatogonia) or the absence of germ cells (Sertoli-cell only, tubular shadow). Different germ cell stages were identified based on their morphology. The number of evaluated tubules per section ranged from 65 to 559 tubules, with an average of 219 tubules per tissue section.

### Evaluation of tissue composition using the point counting approach

For the analysis of tissue composition and the quantification of germ cells, two independent MAGE A4-stained tissue sections (at least 15 µm distance) were evaluated for each individual. Single images of the entire sections were photographed at 10× magnification (7–60 pictures depending on the size of each section) using the ViewPoint software (PreciPoint GmbH). For subsequent analysis, the pictures were transferred to PowerPoint (Microsoft Office 2010, Microsoft Cooperation) and the same grid with 50 randomly distributed points was placed on each image. Each point was evaluated and assigned to one of the following categories: tubule with germ cells, tubule without germ cell, degenerated tubule or interstitium. It is of note that blood vessels, *vas deferens* or hyalinized interstitial structures, were assigned to the category interstitium. The relative proportion of the individual categories in tissue fragments was assessed.

### Quantification of spermatogonial numbers

Spermatogonial quantification was performed in two independent tissue cross-sections to assess the absolute numbers per mm³ (Heckmann *et al.* 2018). The ViewPoint software (PreciPoint GmbH) was used to determine all MAGE A4-positive and -negative spermatogonia within each section. Spermatogonia were identified based on their location, shape and size. In tissues of pre-/pubertal boys, the number of spermatogonia ranged from 212 to 7826 cells and in adult tissues, from 1423 to 3476 cells. In 23 transwomen, spermatogonia were detected and numbers ranged from 58 to 8602 cells. In the tissue from one additional transwoman, only 12 spermatogonia were counted. While this sample was included in the analysis of tissue composition and most advanced germ cell type, it was excluded from further calculations regarding spermatogonial numbers per area. Reason for this is the low number of spermatogonia which may not be representative of the entire tissue due to tissue heterogenity. To determine the number of spermatogonia per mm^2^, the diameter of five nuclei of spermatogonia was measured and revealed a mean diameter of 7.58 µm ± 0.87 µm. The area of each cross-section was measured and ranged from 2.20 to 54.85 mm². The numbers of germ cells per mm³ were calculated using the previously published equation (Gundersen & Jensen 1985, Wreford 1995):


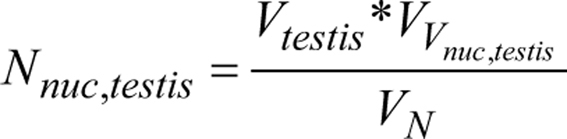



where N_*nuc,testis*_ = spermatogonia (spg) numbers per mm³; *V*_testis_ = mm³; *V*_*V*__nuc__, testis_ = number of spg multiplied by the mean nuclear volume of the respective spg divided by the volume of the evaluated cross-section (area in mm² × 3 µm); *V_N_* = mean nuclear volume.

### Statistical analyses

Analyses were performed using GraphPad Prism® Version 5.0 (GraphPad Software, Inc.). The components of testicular tissue are given as a percentage and were analyzed using the non-parametric Kruskal–Wallis test. Spermatogonial numbers were analyzed by ANOVA and* post-hoc* least significance difference test. Correlations between spermatogonial numbers and hormonal values were determined using the non-parametric Spearman *r* test. Linear regression was used to analyze the relationship between the spermatogonial numbers per mm³ and hormonal values, age or testis weight with 95% CI and calculation of the coefficient of determination *R²*. Significant differences between the groups are marked with * for *P* < 0.05, ** for *P* < 0.01 and *** for *P* < 0.001.

## Results

### Testicular tissue composition in transwomen on the day of GAS

**Figure 1:**
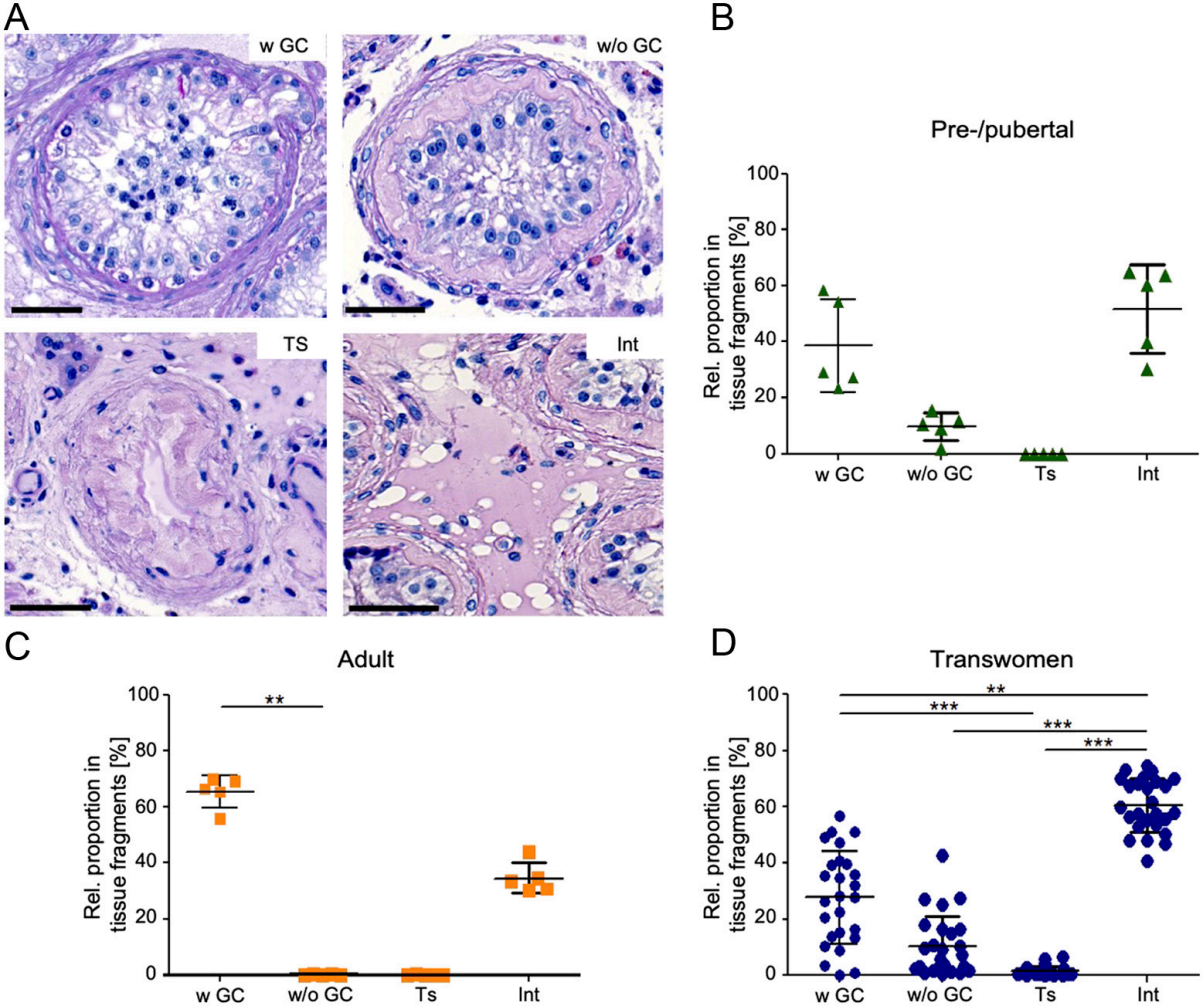
Testicular tissue composition in adult transwomen and control groups. (A) PAS stainings representing the four categories evaluated for the point counting analysis: tubule with germ cells (wGC), tubule without germ cells (without GC), tubular shadow (Ts) and interstitium (Int). The relative proportions of the categories were determined for pre-/pubertal controls (*n*  = 5, B), adult controls (*n*  = 5, C) and transwomen (*n*  = 25, D). Scale bar: 50 µm. Graphs show mean ± s.d. Data were analyzed by non-parametric Kruskal–Wallis test and significant differences between the categories are marked with ^**^P < 0.01 and ^***^P < 0.001.

Analysis of the testicular tissues of transwomen regarding the most advanced germ cell type per tubule revealed suppressed spermatogenesis in all subjects included. Round spermatids were only observed in three transwomen (Supplementary Fig. 1, see section on supplementary materials given at the end of this article), while meiotic arrest at spermatocyte stage was found for five transwomen. Around 17 transwomen had spermatogonia as the most advanced germ cell type in their testicular tissues (Supplementary Fig. 1B). Further analysis focused on the tissue composition; for this, the relative proportions of tubules with and without germ cells, tubular shadows and interstitium were determined (Fig. 1A) and compared to the control groups. In the pre-/pubertal control group (Fig. 1B), the interstitium was the dominant compartment of the testes (51.7 ± 15.7 %), followed by tubules with germ cells (38.5%). The adult control tissues consisted largely of tubules with germ cells (65.3 ± 5.6%) and 34.6% interstitium (Fig. 1C). In testicular tissues of transwomen, the composition differed from adult control testes (Fig. 1D), as the interstitium accounted for 60.4 ± 9.5% and tubules with germ cells only for 27.7 ± 16.6%. In comparison to the adult control group, the proportion of tubules with germ cells was significantly decreased in testicular tissues of transwomen (*P* < 0.01), whereas the proportions of tubules without germ cells and interstitium were significantly increased (*P* < 0.01).

### Spermatogonial numbers in testicular tissues of transwomen on the day of GAS

**Figure 2:**
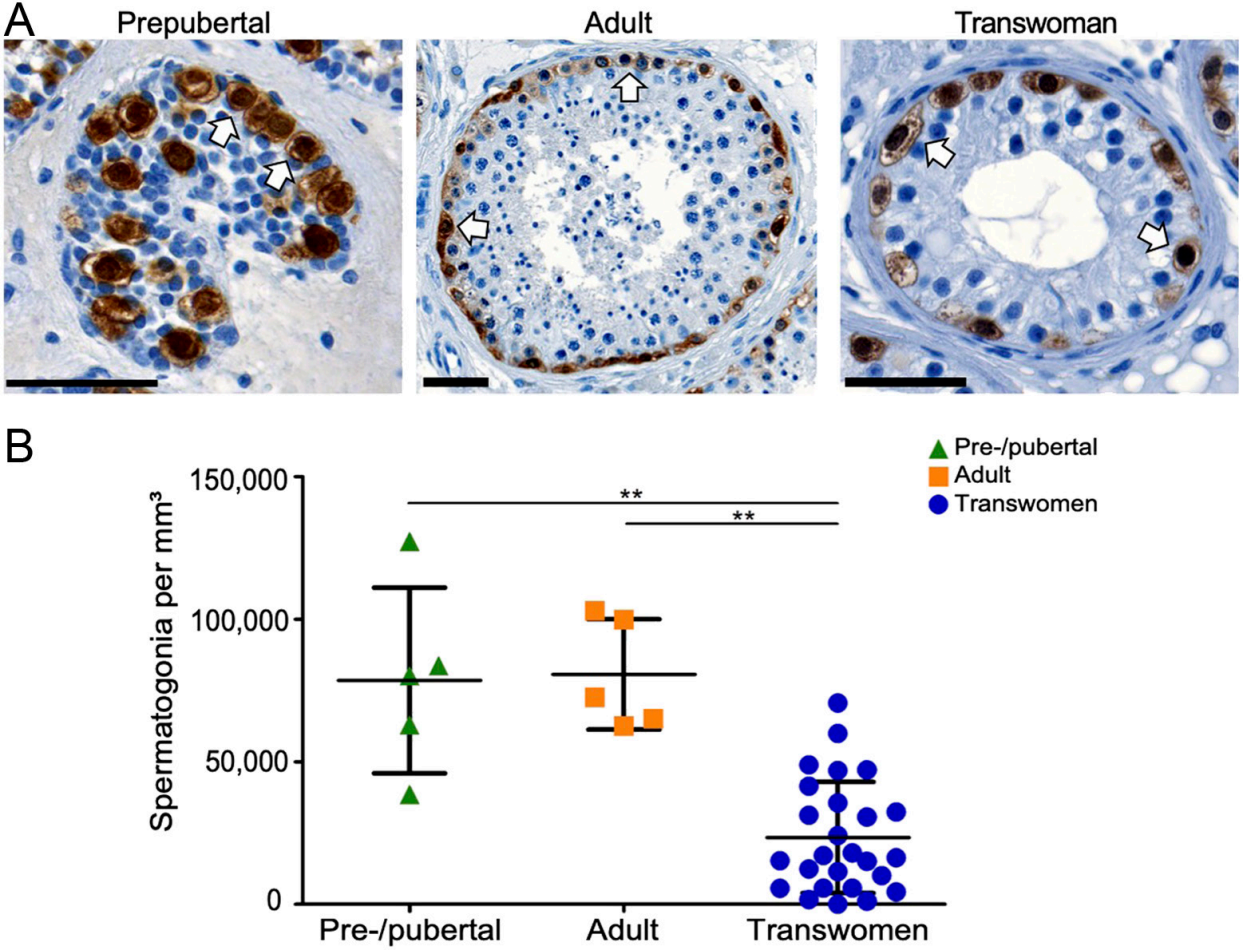
Detection and determination of absolute spermatogonial numbers in testicular tissues of transwomen and control groups. (A) Images show immunohistochemically stained testicular tissue sections with MAGE A4-positive spermatogonia (white arrows). Scale bars = 50 µm. (B) Spermatogonia were counted in two independent tissue sections per patient for the pre-pubertal control group (*n*  = 5), the adult controls (*n*  = 5) and the transwomen group (*n*  = 24). Absolute spermatogonial numbers per mm³ were calculated for the three groups and analyzed by ANOVA and non-parametric Kruskal–Wallis test and significant differences between the categories are marked with ^**^ *P* < 0.01.

Spermatogonia were counted in MAGE A4-stained tissue sections of transwomen and controls. The testicular tissues of pre-/pubertal controls contained on average 78.5 ± 32.5 spermatogonia/mm^3^ (×10³) (range from 38.6 to 127.3 spermatogonia/mm^3^ (×10³)) (Fig. 2B). In the adult control group, the mean spermatogonial numbers were similar at 80.6 ± 19.4 spermatogonia/mm^3^ (×10³) (range 62.7 to 104.0 spermatogonia/mm^3^ (×10³)). Transwomen, however, showed significantly lower spermatogonial numbers compared to both control groups (*P* < 0.01). Testicular tissues of transwomen contained on average 25.1 ± 19.4 spermatogonia/mm³ (×10³) with a high inter-individual variability as reflected by the range from 0.0 to 70.6 spermatogonia/mm³ (×10³) (Fig. 2B). Spermatogonial numbers were not significantly different with regard to the continued or non-continued GATH prior to surgery.

### Association of spermatogonial numbers in transwomen with hormone values

**Figure 3:**
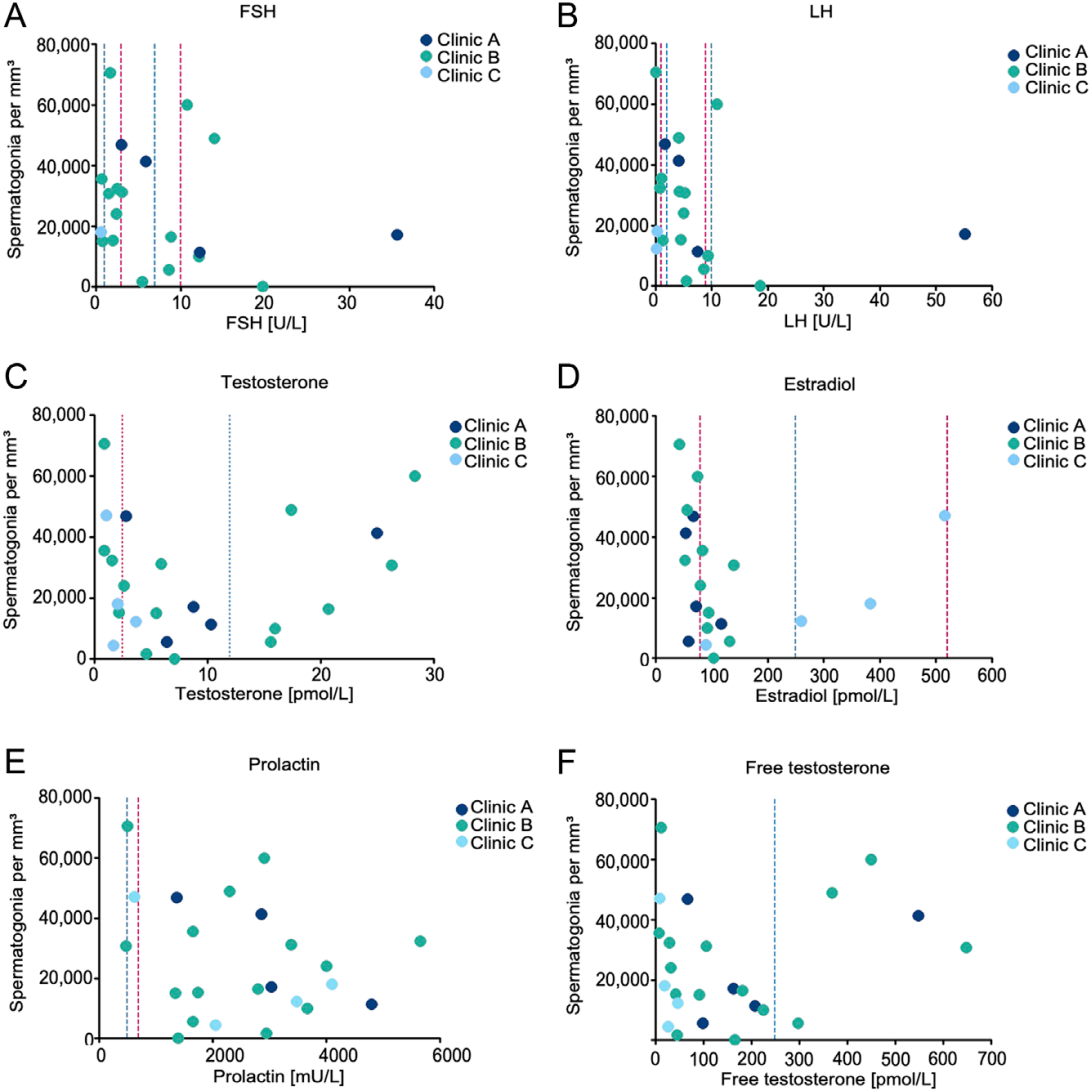
Correlations between the spermatogonial numbers and the blood serum hormone values of transwomen. Spermatogonia per mm³ were correlated with levels of (A) follicle-stimulating hormone (FSH) (*n*  = 23), (B) luteinizing hormone (LH) (*n*  = 22), (C) testosterone (*n*  = 24), (D) estradiol (*n*  = 24), (E) prolactin (*n*  = 23) and (F) free testosterone (*n*  = 24). Spearman's rank correlation coefficient analysis was performed and revealed significant correlations for LH and estradiol. The blue lines mark the male reference values and the red lines mark the female reference values. Clinic A (*n*  = 5) stopped the gender-affirming hormone therapy 4–6 weeks prior to the surgery, clinic B (*n* = 16) stopped the hormone therapy 2 weeks prior to the surgery, while clinic C (*n* = 4) continued the therapy until the day of GAS.

Correlations between spermatogonial numbers in transwomen were established to assess the impact of hormones (Fig. 3). Irrespective of the pre-treatment in the different clinics, no significant correlations were observed between serum hormone values for FSH, LH, testosterone, estradiol, prolactin or free testosterone (Fig. 3A, B, C, D, E and F). However, a significant correlation of spermatogonial numbers and serum hormone values was observed for transwomen treated in clinic B for LH (Fig. 3B; *P* < 0.05) and estradiol (Fig. 3D; *P* < 0.001). A linear regression analysis of estradiol values and spermatogonial numbers for transwomen of clinic B revealed that significantly less spermatogonia were present (*P* < 0.05) in testes of individuals with higher estradiol levels.

### Testicular weight and age of the included individuals

**Figure 4:**
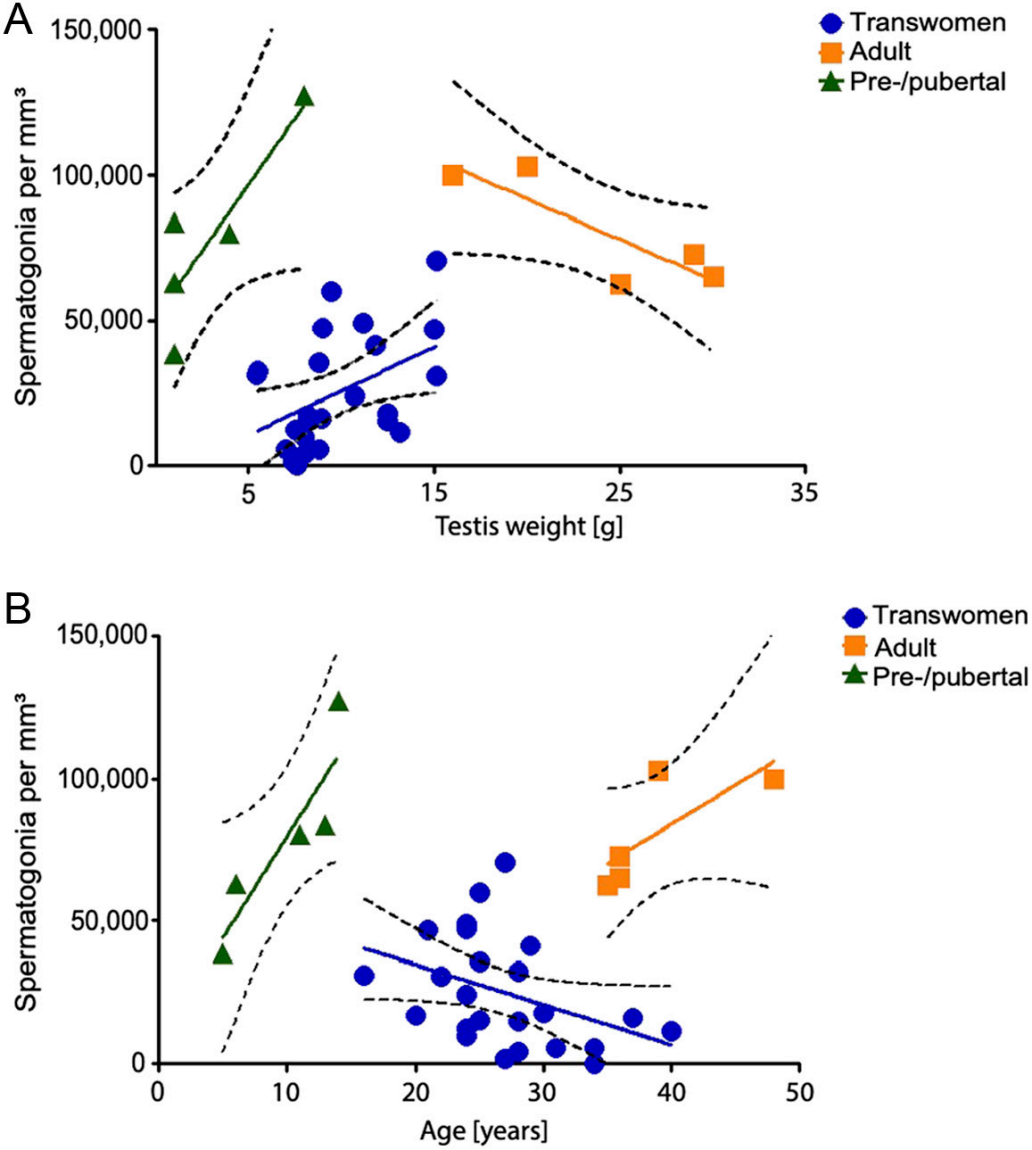
Linear regression of relationships between the spermatogonia per mm³ and the testis weight (A) or age (B) on the day of surgery. Spermatogonial numbers and testis weight or age were plotted for the groups of pre-/pubertal (*n*  =5), adult (*n*  = 5) and transwomen (*n*  = 24). Statistically significant linear regressions were found for the testis weight in pre-/pubertal and transwomen group (*P* < 0.05).

Comparing the effect of age and testis size on spermatogonial numbers revealed three separated subgroups depending on the developmental status (pre/pubertal/adult/transwomen) with transwomen being intermediate (Fig. 4A). Spermatogonial numbers in testes from transwomen correlated with testis size (*P* < 0.05). An inverse correlation with age was found for spermatogonial numbers (*P* < 0.05). However, spermatogonial numbers neither correlated with the duration of the hormone therapy nor the dose of the estrogens taken (Supplementary Fig. 2).

## Discussion

Counseling transwomen with regard to fertility options is a necessity, as they lose their fertility irreversibly at the time of GAS. If sperm were not preserved prior to or during GAHT treatment, this surgery is the last chance to preserve remaining germ cells present in the testicular tissues. If only undifferentiated germ cells remain, testicular tissues could be included in fertility preservation programs using protocols established for tissues from pre-pubertal cancer patients (Goossens *et al.* 2020). Protocols for cryopreservation are distinct from those used for sperm (Goossens *et al.* 2020) and have been established for oncology patients (ANDROPROTECT®); however, the derivation of sperm from these cryopreserved spermatogonia has only been achieved in animal models so far (Neuhaus & Schlatt 2019). As protocols that are currently under development to derive sperm from these immature testicular tissues require the presence of sufficient numbers of spermatogonia, we investigated the numbers of spermatogonia per mg testicular tissue of transwomen following GAHT in comparison to pre-pubertal and adult individuals.

We found that 17 out of 25 (68%) transwomen tissues contained spermatogonia as the most advanced germ cell type. This finding is in line with studies reporting an arrest at spermatogonial level in 80 and a maturation arrest in 36.4% of the examined testicular tissues (Jindarak *et al.* 2018, Matoso *et al.* 2018). In these cases, cryopreservation of testicular tissues to preserve the spermatogonia constitutes the only option to preserve fertility.

Importantly, we found high inter-individual differences with regard to spermatogonial numbers ranging from 0 to 70^5^ spermatogonia per mm^3^ testicular tissue. In comparison with the number of spermatogonia in adult and pre-/pubertal testes, spermatogonial numbers were significantly lower in transwomen. If, however, there is a sufficient amount of spermatogonia in the testicular tissue of transwomen, cryopreservation of tissues should be considered for reproductive purposes. It needs to be considered, however, that in contrast to pre-pubertal boys, for whom only a small biopsy is cryopreserved as fertility reserve, both testes can be cryopreserved for transwomen, so that the absolute number of spermatogonia is at least as high as for pre-pubertal boys. Intriguingly, our findings suggest that GAHT not only leads to a reduction of differentiated germ cell numbers but also to a reduction of the spermatogonial population. This finding is generally consistent with previous reports describing small cohorts (between 4 and 11 subjects), which observed a reduction in the spermatogonial population (Lu & Steinberger 1978, Sapino *et al.* 1987, Schulze 1988), but no absolute numbers were available in these studies for comparison. These results, however, need to be expanded in the future with immunohistochemical stainings and quantification of undifferentiated spermatogonial subpopulations (i.e. promyelocytic leukemia zinc finger (PLZF)) and for proliferating spermatogonia (i.e. antigen KI-67) to uncover the influence of GAHT in-depth. The loss of germ cells might be caused by direct effects of GAHT on the spermatogonia or indirect effects due to suppressed testosterone and gonadotropin levels. Both explanations are conceivable as a reduction of spermatogonia was reported after treatment with estrogens only (Schulze 1988) as well as with CPA only (Schulze 1978). After performing single cell analyses of testicular tissues from two transwomen, a much lower proportion or even absence of differentiating spermatogonia, spermatocytes and spermatids was found (Guo *et al.* 2020). This dataset also suggests a high variability between samples and the need to correlate findings with clinical data (i.e. treatment duration, type of GAHT) in a larger cohort.

Our study underlines that the daily CPA dose, the duration of treatment and the dosage of estrogens are not decisive for spermatogonial numbers. This observation suggests variable effects of the therapy on the testis or an individual sensitivity to the therapy. This is in line with observations fromLeavy *et al.* (2017) who reported transwoman with intact spermatogenesis after 6 years of treatment with estrogens and CPA. It remains to be assessed if different pre-operation hormone treatments lead to similar results compared to those reported in this study, as our group was rather homogenous. Moreover, longitudinal studies have to performed examining the effects of GAHT on spermatogenesis and steroidogenesis with different treatment regimens. Furthermore, studies have to be performed using age-matched pre-pubertal/pubertal and adult subjects to confirm our findings.

We detected other factors, however, that could serve the treating clinician as predictors of the testicular status. Serum LH and estradiol levels of transwomen who stopped the GAHT 2 weeks prior to surgery (clinic B) were negatively correlated with spermatogonial numbers. High LH levels above the male reference values indicate low spermatogonial numbers and impaired spermatogenesis, an observation that is in line with data from Klinefelter patients, reporting a higher chance to isolate sperm in individuals with LH levels ≤17.5 U/L (Rohayem *et al.* 2015). An additional predictive factor for the presence of spermatogonia is the testicular size. Our analysis revealed a linear regression of testis weight and spermatogonial numbers of transwomen, with more spermatogonia present in those testes with a higher weight. For clinicians, an orchidometer or ultrasound will help to determine the testicular volume. Furthermore, we want to stress that spermatogonial numbers were not different with regard to pre-surgical treatment regimens, but feminized blood serum levels were seen when treatment was not stopped. Stopping GAHT is done, because of the fear of side effects during surgery, this concern was found to be unsubstantiated however, based on a recent publication (Kozato *et al.* 2021).

Our data underline that transwomen should be counseled with regard to fertility preservation prior to the start of hormonal treatment when the chance of cryopreserving sperm is highest. In case fertility preservation was not addressed prior to treatment, our data suggest that testicular tissues containing spermatogonia can be preserved at the time of GAS, although their numbers are reduced. Importantly, the presence of spermatogonia and the spermatogenic state in transwomen can be predicted prior to surgery by measurements of serum hormone values and determination of testes weight.

## Supplementary Material

Supplementary Figure 1

Supplementary Figure 2

## Declaration of interest

There is no conflict of interest that could be perceived as prejudicing the impartiality of the research reported.

## Funding

This work was supported by a research stipend of the German Society of Andrology (DGA) to Florian Schneider in 2017.

## Author contribution statement

Conception and design: NN, FS, JW, SK, SS; data acquisition, analysis, interpretation: JD, NN, FS, JW; article drafting: JD, FS, JW, NN. All authors participated in the revision and approved the final submitted version.
