# Supplementary figures and images for: New perspectives on fertility in transwomen with regard to spermatogonial stem cells
Source: Reprod Fertil. 2023 Jan 18;4(1):e220022. doi: 10.1530/RAF-22-0022 (PMC9874957; doi:10.1530/RAF-22-0022)

A

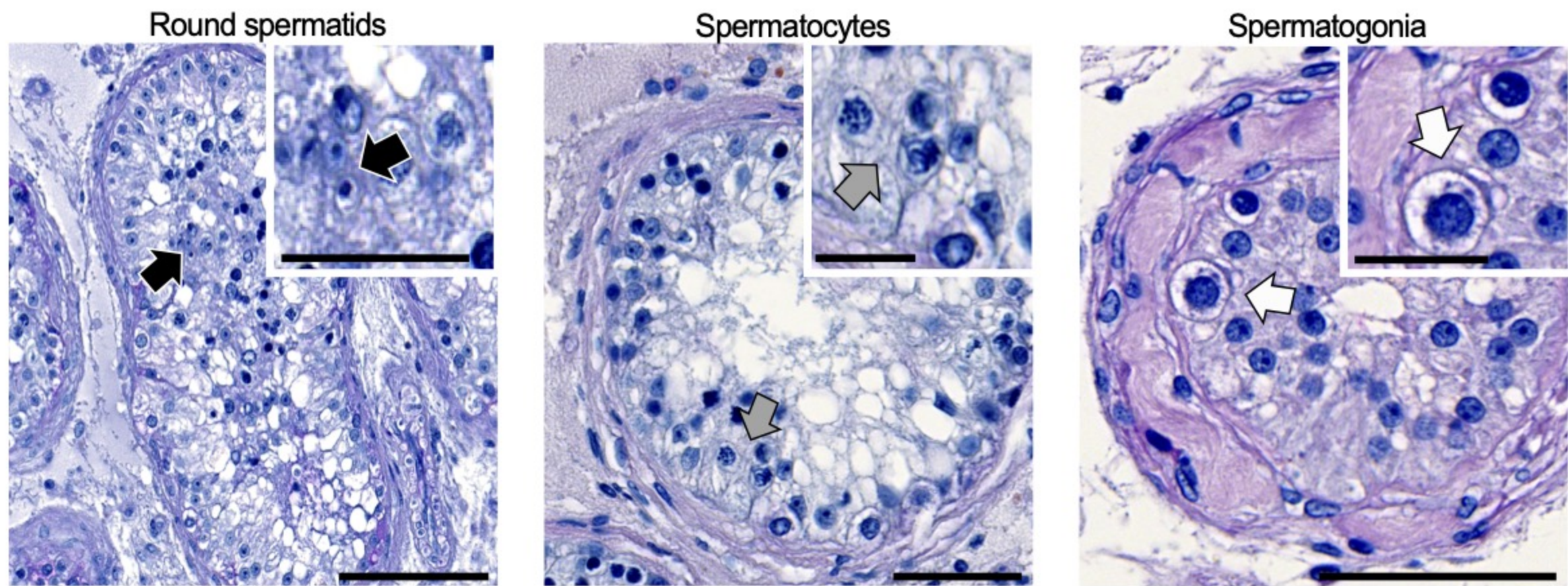

B

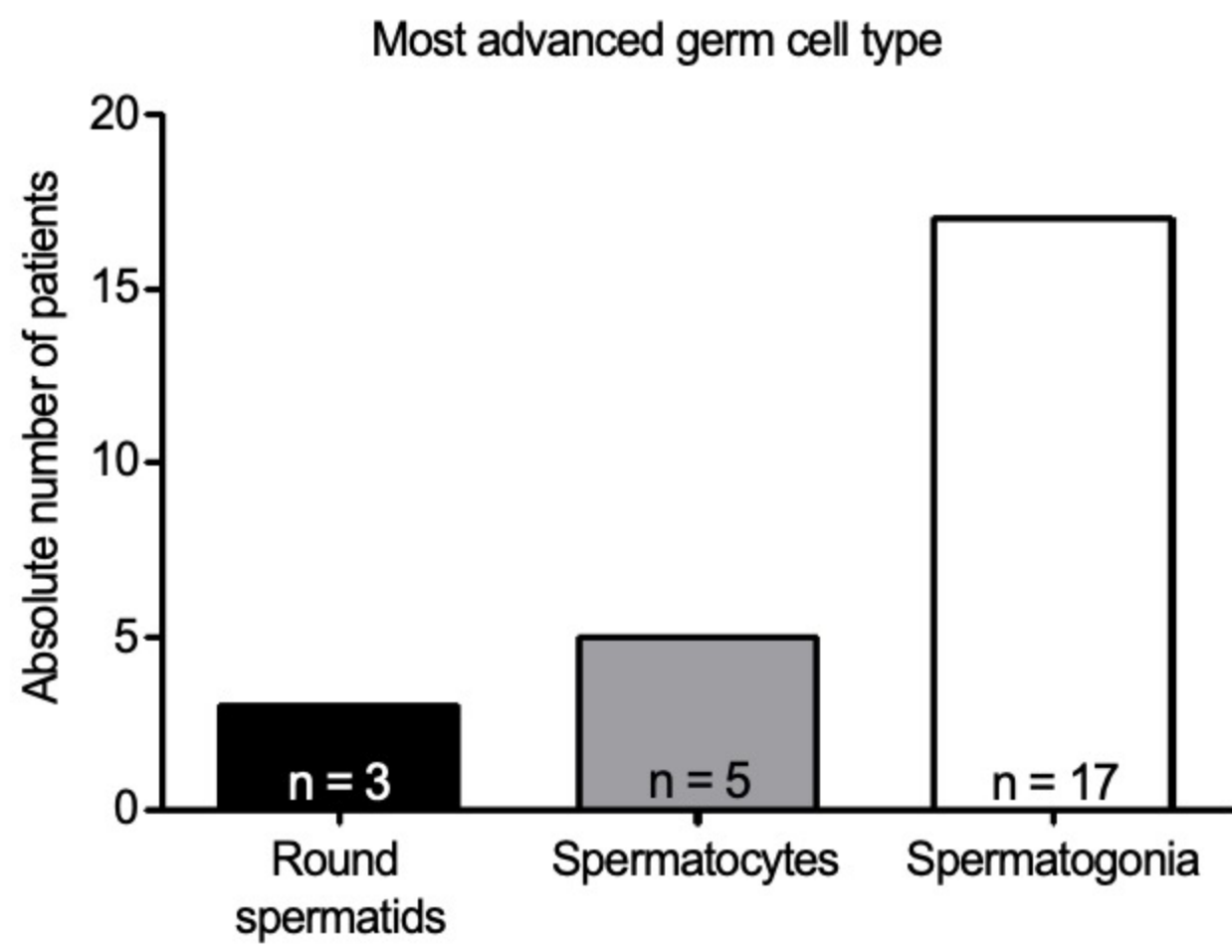

Supplement: Supplementary Figure 1 [file supplementary_figure_1.pdf]

A

Dose of estrogens

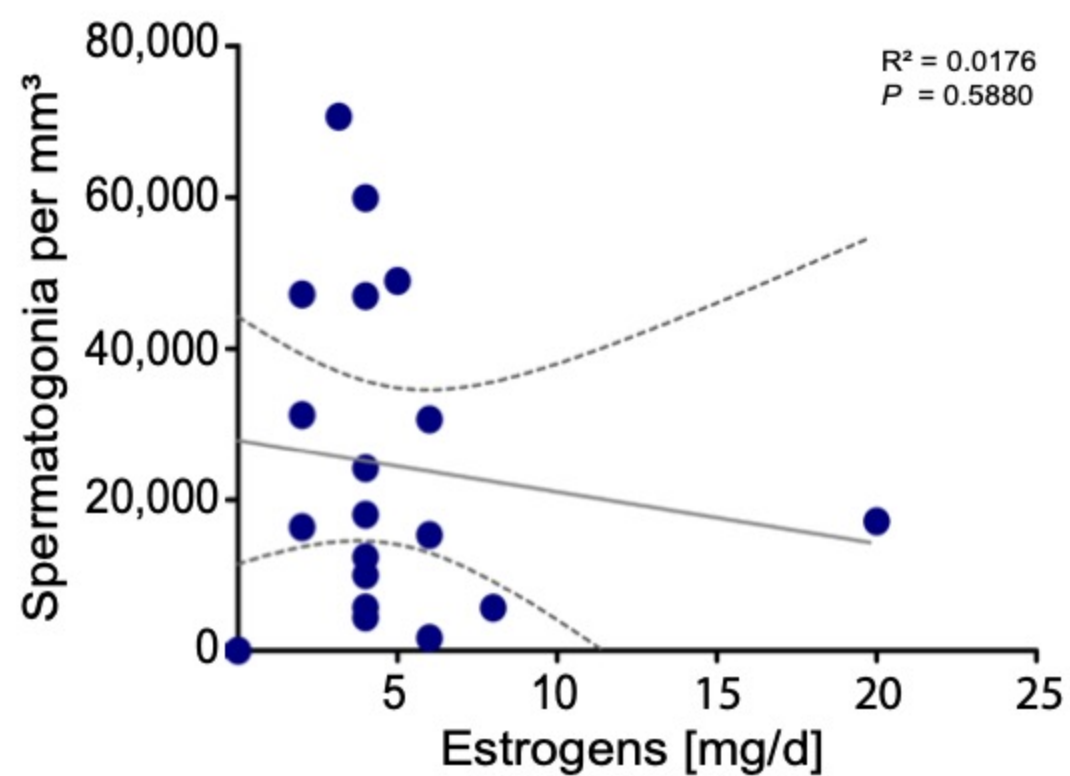

B

Duration of hormonal treatment

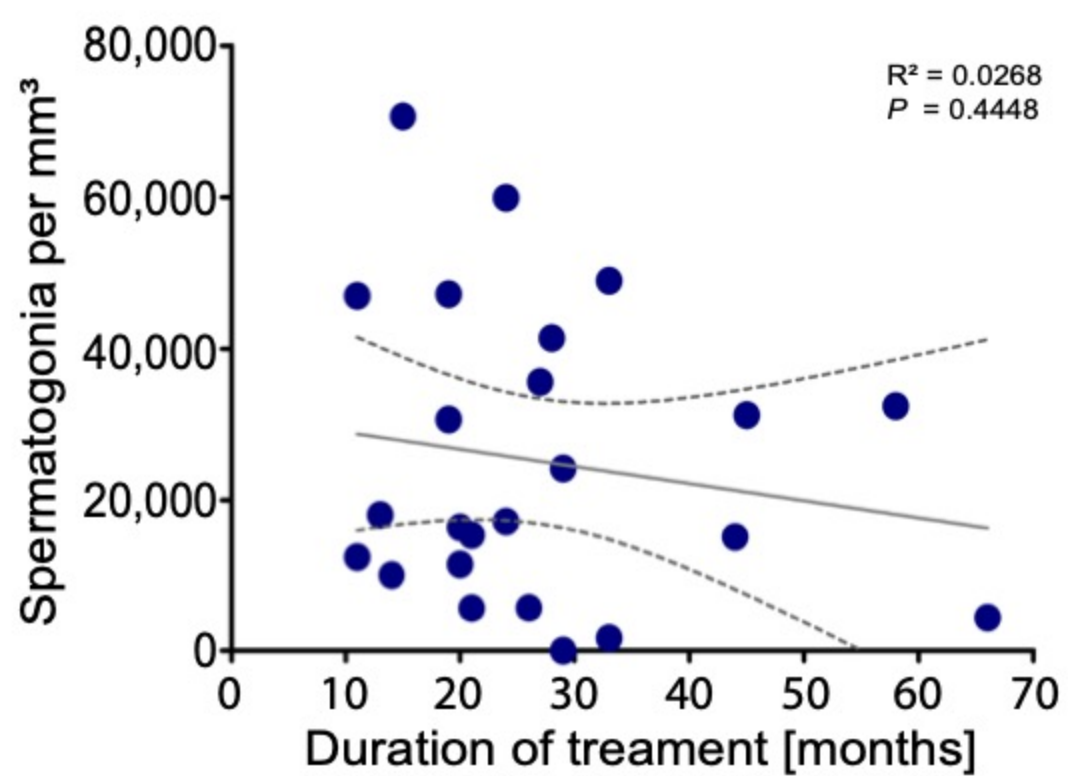

Supplement: Supplementary Figure 2 [file supplementary_figure_2.pdf]
